# The incidence of hip fractures in Norway –accuracy of the national Norwegian patient registry

**DOI:** 10.1186/1471-2474-15-372

**Published:** 2014-11-13

**Authors:** Mikkel P Høiberg, Jeppe Gram, Pernille Hermann, Kim Brixen, Glenn Haugeberg

**Affiliations:** Department of Internal Medicine, Hospital of Southern Norway, Egsveien 4, Kristiansand, N-4604 Norway; Institute of Clinical Research and OPEN Odense Patient Data Exploritative Network, University of Southern Denmark, Odense, Denmark; Department of Endocrinology, Hospital of Southwest Denmark, Esbjerg, Denmark; Department of Medical Endocrinology, Odense University Hospital, Odense, Denmark; Department of Rheumatology, Hospital of Southern Norway, Kristiansand, Norway; Faculty of Health and Sport Sciences, University of Agder, Kristiansand, Norway

**Keywords:** Validation studies, Epidemiology, Incidence, Hip fracture

## Abstract

**Background:**

Hip fractures incur the greatest medical costs of any fracture. Valid epidemiological data are important to monitor for time-dependent changes. In Norway, hip fractures are registered in the Norwegian Patient Registry (NPR), but no published national validation exists. The aim of the present study was a national validation of NPR as a register for hip fractures using diagnostic codes (ICD-10 S 72.0-2) and/or procedure codes (NOMESCO version 1.14 NFBxy (x = 0-9, y = 0-2) or NFJxy (x = 0-9, y = 0-2).

**Method:**

A nationwide, population-based cohort comprising a random sub-sample of 1,000 hip fracture-related entries for the years 2008–09 was drawn from the NPR. 200 entries were defined by a combination of diagnostic and procedure codes (subsample 1), 400 entries were defined by diagnostic codes only (subsample 2) and 400 entries were defined by procedure codes only (subsample 3). Accuracy was ascertained through comparison with discharge summaries, procedure notes and X-ray reports requested from 40 health institutions. Comparisons between groups were done by chi^2^ for categorical and t-test for continuous variables.

**Results:**

792 health records from 32 institutions were reviewed. High accuracy (98.2%, 95% C.I. 96.5-99.9%) was found for subsample 1, a combination of diagnostic and procedure codes. Coding errors were prominent in other subsamples. Defining fractures by a combination of diagnostic and procedure codes, annual average hip fracture incidence in Norway was 9,092 (95% C.I. 8,934 -9,249), excluding only 6.5% of all hip fractures defined by wider definitions.

**Conclusions:**

Based on current coding practice in Norway, a reliable national estimate of hip fracture incidences is found by a combination of relevant ICD-10 and NOMESCO codes in the NPR. This method may be used for monitoring epidemiological changes.

**Electronic supplementary material:**

The online version of this article (doi:10.1186/1471-2474-15-372) contains supplementary material, which is available to authorized users.

## Background

In Scandinavia, about 40% of women and 13–23% of men will experience a fracture after the age of 40 years and hip fractures comprise approximately half of these [1]. In 2005, hip fractures were the cause of 60–70% of all fracture-related hospital admissions for Swedish citizens aged 50+ years [2]. Hip fractures incur the greatest medical costs of any fracture [3, 4], thus valid epidemiological data are important to understand the magnitude of the problem and to monitor changes over time.

In the Scandinavian countries, all personal hospital records and registrations in databases are unequivocally identified by personal registration numbers. In Norway, hip fracture registration is done in the Norwegian Patient Registry (NPR) based on both the International Statistical Classification of Diseases and Related Health Problems (ICD) by the World Health Organisation (WHO) [5] and Nordic Medico- Statistical Committee (NOMESCO) version 1.14 procedure codes [6]. Entries in clinical patient databases such as NPR can be used for epidemiological surveillance of disorders such as hip fracture, but due to coding errors validity may vary on a local [7] and national level [8].

For hip fractures as of today, no national validation of fracture incidences for NPR exists, even though several local or regional fracture-cohort examinations have shown that ICD-coding can be incorrect [9–13]. In Denmark, Vestergaard *et al.*[14] examined a small random sample of fracture reports registered by ICD-codes from a national database and found the accuracy of fracture reports to be as high as 97%. Using data from three Finnish hospitals, the accuracy of pertrochanteric fractures in the Finnish Hospital Discharge Register was found to be high at 96% [15]. A study from Norway, exploring the same research question by using a regionally derived fracture database and comparing these data with registries in NPR [16], suggested the use of ICD-coding for fracture definition alone and contrasted an earlier Norwegian regional study reporting an overestimation of fracture incidence rates of 19% by this method [11]. Due to previously discrepant findings, there is a need for easy methods for reliable epidemiological surveillance of especially resource demanding diseases like hip fracture.

In this study, we therefore aimed to validate the registration of hip fractures recorded in the NPR on a national level. Furthermore, we aimed to identify sources of error in NPR with respect to hip fracture data and finally to develop a simple but reliable method for estimating hip fractures incidences in Norway based on hip fracture registrations in NPR.

## Methods

### Study design

The study was nationwide, population-based and comprised a randomly selected subsample of 1,000 hip fracture-related entries in NPR, equally divided between the years of 2008 and 2009. Accuracy of the registered NPR entries was ascertained through comparison with a combination of event-specific discharge summaries, surgical procedure notes and X-ray reports. The Norwegian background population comprised 4.8 million citizens as of 2009 [17].

### Sources of data

Data on civil registration number, ICD-10 and procedure code, name of health institution/hospital, department responsible for treatment as well as admission and discharge dates were retrieved from the NPR of Norway. Data on national hip fracture incidence rates defined by ICD and NOMESCO procedure codes for the years 2008 and 2009 were also acquired from NPR.

During the examined period, hip fractures were treated in 40 different hospitals. All heads of departments responsible of treatment were contacted by letter and telephone and informed about the study. Copies of discharge summaries, descriptions of performed surgical procedures from health records and X-ray reports were requested. Non-respondents were issued a reminder approximately six weeks after the initial contact.

Hip fractures were defined as uniformly described fractures in both x-ray reports and health records, regardless of choice of treatment. All records were examined by MH for fulfillment of this fracture definition.

### Identification of hip fracture cohort

**Figure 1:**
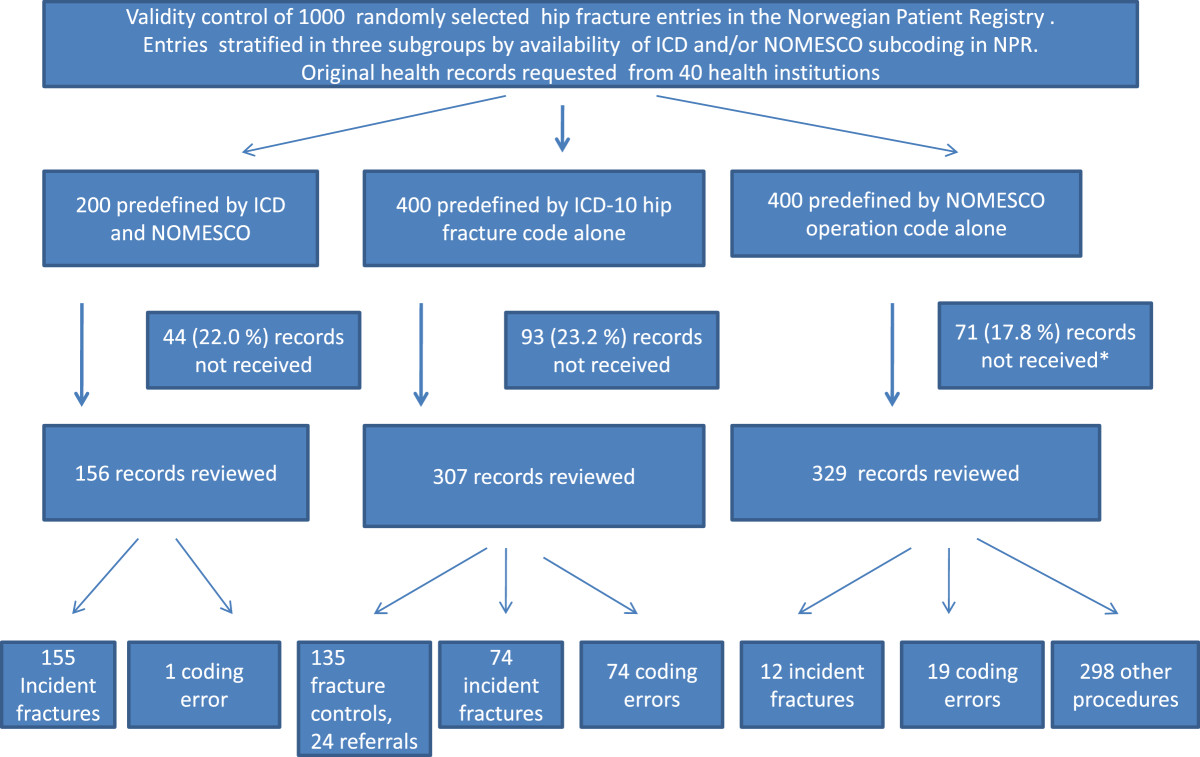
**Flow chart of study design and sub-grouping of examined health records identifying patients with hip fracture in the Norwegian patient registry (NPR).** *No significant differences in rates of received health records between groups (p = 0.395).

The 1,000 hip fracture related events from NPR were divided into three subsamples: 200 events defined by a combination of ICD-10 DRG-codes s72.0-72.2 in combination with NOMESCO version 1.14 procedure codes [6] NFBxy (x = 0-9, y = 0-2) og NFJxy (x = 0-9, y = 0-2) (subsample 1), 400 events defined by ICD-code without NOMESCO code (subsample 2) and 400 events defined solely by NOMESCO code without ICD-code for hip fracture (subsample 3). Readers unfamiliar with these coding systems are referred to Additional file 1 for further information. Hip-fractures by ICD-coding were assumed to be treated by an orthopedic surgeon and thus registered together with NOMESCO operation codes. Missing NOMESCO codes were expected in case of conservative treatment of small, stable fractures, patients experiencing death before operation or due to coding failure, whilst missing ICD-10 codes could be seen with other diseases treated with the same kind of intervention (e.g. arthrosis of the hip) or due to coding failure. Assuming that fractures defined by a combination of fracture codes and procedure codes had a higher validity than fractures defined by only one of these coding systems (as two coding-errors in the same patient, all other factors being equal, are less likely to occur than a single coding error in the same patient), the subsamples had unequal sample sizes. A gross overview of study design and sub-grouping of examined health records is shown in Figure 1.

### Ethics

The study was approved by the Regional Ethics Committee of South-Eastern Norway (REK Sør-øst B reference number: 2009/1809b).

### Informed consent

The purpose of this study was solely to validate recent historical hip fracture entries in the NPR and estimate national hip fracture incidence-rates. Therefore, the Regional Ethics Committe of South-Eastern Norway granted access to medical records for this purpose without personal informed consent from the patients in question (REK Sør-øst B reference number 2009/1809b).

### Statistics

Descriptive data are presented as total number, proportion or incidence rates and confidence intervals, as applicable. Confidence intervals for proportions were derived using Wilson’s method. Comparisons between groups were done by chi^2^ for categorical and t-test for continuous variables.

## Results

A total of 792 (79.2%) individual health records from a total of 32 institutions were received, 404 (51.0%) of these were received within the first six weeks. A total of eight institutions covering 208 (20.8%) requested health records did not respond. Analysis for non-response bias by comparison of gender and age at hospital admission showed no significant differences between responding and non-responding health institutions. Furthermore, no significant differences between rates of received health records in the below defined subsamples 1–3 were found (p = 0.40).

In the subsample 1 defined by a combination of ICD-10 fracture and NOMESCO procedure code in NPR, a total of 156 were received (78.0% response rate). One of these could not be verified in the original health records and comprised a false positive registration. In a single case, the Girdlestone procedure (NOMESCO: NFG09) was performed according to the case notes while NFB/NFJ procedure codes had been entered into NPR. Thus, the validity of hip fractures defined from both ICD-10 and NOMESCO codes was 99.4% (95% C.I. 96.5-99.9%).

**Figure 2:**
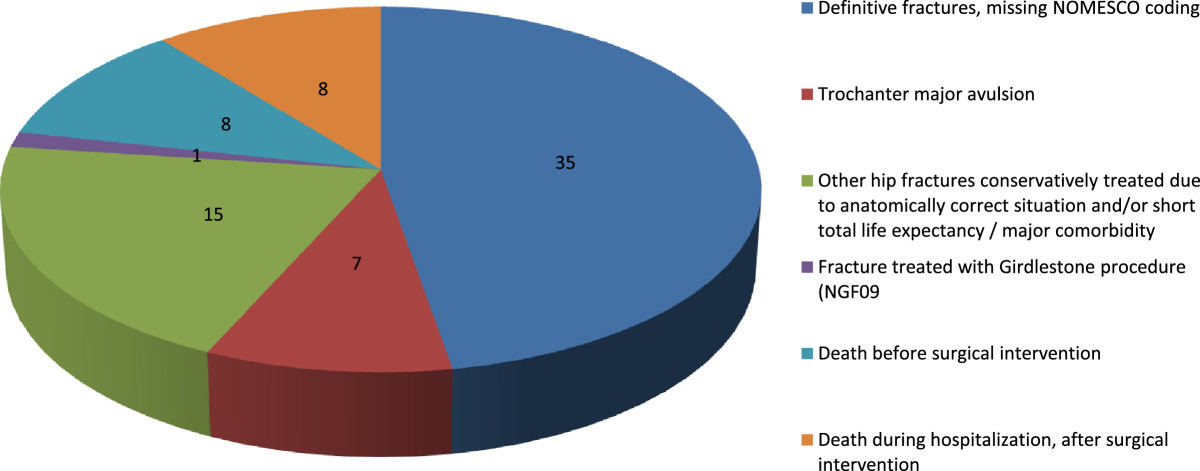
**True hip fracture-entries in the Norwegian Patient Registry (NPR) defined by ICD-code for hip fracture alone and no procedure code indicative of hip fractures.** 74 fractures (24.1% 95% C.I. 19.7-29.3%) in subgroup 2 that would have been missed by using a narrow hip fracture definition defined by a combination of both ICD and NOMESCO.

In the subsample defined by ICD-code alone, data on 307 out of 400 were received (76.7% response rate). A substantial part of this subsample consisted of follow-up controls after hip fracture (135 out of 307). However, a total of 74 (24.1% 95% C.I. 19.7-29.3%) false positives were found during validation by the use of local health records, as shown in Table 1.Furthermore, this group also included 74 fractures (24.1% 95% C.I. 19.7-29.3%) that would have been missed by using a narrow hip fracture definition defined by a combination of both ICD and NOMESCO code, as seen in Figure 2.

**Table 1:** **Entries in the Norwegian Patient Registry defined by ICD-10 alone and no procedure codes indicative of hip fracture**

Subgroup defined by ICD-10 alone	True ICD-code	Number of cases
Validated reason for hospital stay		
Outpatient control		135
Transfers for rehabilitation at other health institutions		24
*Transfers for rehabilitation at other health institutions*		14
Outpatient control, coding error (13 in total, specified below in brackets)		
**Definitive fractures with performed operations, missing NOMESCO coding**	S 72	35
**Conservatively treated fractures**	S 72	15
*Erroneous registration, no contact*		15
**Death before operation for hip fracture**	S 72	8
**Death after operation**	S 72	8
*Admission in an internal medicine ward, no current fracture*	-	7
**Trochanter major avulsion, conservative treatment**	S 72	7
*Hip contusion, no fracture (1)*	S 70.0	7
*Tibial fracture (3)*	*S 82*	*5*
*Removal of osteosynthesis material as a manner of pain-reduction in hip fractures treated >1 year prior to actual hospital stay*	Z47.0	3
*Humerus fracture (1)*	S 42.2	3
*Prosthetic failure or luksation (1)*	T 84.0	3
*Lower leg pain or hip fracture >1 year prior (3)*	-	3
*Dupyutrens contracture (2)*	M 72.0	2
*Femoral shaft fracture (1)*	S 72.3	2
*Pelvis fracture (1)*	S 32.8	2
**Girdlestone replacement**	S 72	1
*Traumatic vertebral fracture*	S 32.2	1
*Sternum fracture*	S22.0	1
*Distal radius fracture*	S52.5	1
*Quadriceps rupture*	S76.1	1
*Bimalleolar fracture*	S82.5	1
*Cutaneous lesion in the hip region, fine-needle aspiration*		1
*Observation, other reason*	Z03.9	1
*Decubital wound, operation for hip fracture four months prior*	L89	1

*Italic* letters: false positive fracture cases, 74 out of 307 entries.

**Bold** letters: false negative fracture cases (using a strict fracture definition defined by a combination of ICD-10 diagnosis codes and NOMESCO procedure codes), 74 out of 307 entries.

In the final subgroup from the NPR database sample, defined by means of procedure-codes NFBxy (x = 0-9, y = 0-2) or NFJxy (x = 0-9, y = 0-2) without concurrent ICD-codes for hip fracture, data were available on 329 (82.2%). As illustrated in Table 2, 297 (90.3%) of these were “coxarthrosis” treated with mostly hemi-prosthetics, whilst six entries concerned removal of osteosynthesis material or reoperation due to prosthetic failure, five could not be verified in hospital health records, four were secondary prosthetic replacements due to necrosis of the femoral head, and one distal femoral fracture, one epiphysiolysis and one operation for a chondrosarcoma were identified, respectively. Erroneous coding was found in a total of 19 patient records (5.8%, 95% C.I.:3.6-9.0%).

**Table 2:** **Entries in the Norwegian Patient Registry (NPR) defined by NOMESCO procedure codes alone without concurrent ICD-codes for hip fracture, numerical summation of subsample**

Subgroup defined by NOMESCO coding alone	Number
Validated reason for hospital stay (n = 329)	
Coxarthrosis	297
Not identifyable	7
Traumatic necrosis of femoral head	4
Hip fracture not coded with ICD, miscoded	4
Removal of osteosynthesis material or reoperation due to failure	4
Lower leg fracture, miscoded	3
Distal femoral fracture	3
Knee replacement, miscoded	1
Humerus fracture, miscoded	1
Multi-trauma, no hip fracture, miscoded	1
Peri-prosthetic fracture	1
Outpatient control, no operation, miscoded	1
Epiphysiolosis	1
Chondrosarcoma operation with hip prothetics	1

A total of 12 out of 329 NPR registries in this group were fractures that would not have been identified using a narrow fracture definition with a combination of ICD and NOMESCO codes alone due to erroneous ICD-coding.

### Estimation of true hip fracture incidence

Assuming previously proven good coverage of NPR data, we based the estimation of the hip fracture incidence upon the subgroup defined by a combination of both ICD and NOMESCO code, since the accuracy was 96.5-99.9%in this group. For the years 2008 and 2009, a total of 9,359 and 9,157 hip fractures by this definition could be allocated to a civil registration number in the NPR database, respectively. Using these crude numbers, the annual average hip fracture incidence in Norway was 9,092 (95% C.I. 8,934 to 9,249), equaling an overall incidence rate of 189.4 per 100.000 person years. In addition, for the abovementioned years, a total of 1,569 and 1,797 entries in NPR (yearly average of 1,683) were defined by ICD-10 code alone. As the randomly selected subsample of 307 of these identified another 74 (24.1%, 95% C.I. 19.6-29.2%) fractures unaccounted for in the calculated incidence above, this equates to another 393 (321–491, 95% C.I) yearly incident fractures, using Wilsons procedure for confidence intervals of proportions.

Finally, another 12 out of 329 (3.6%, 95% C.I. 1.9-6.5%) fractures could be verified in the group identified by relevant NOMESCO codes alone. The average yearly civil registration number-connected incidence of this category in NPR was 6,610 – thus the identified fractures would amount to another estimated 241 (131–414, 95% C.I.) fractures yearly.

As a whole, the best estimate of hip fractures in Norway for the years 2008–2009 is 9,726 (9,092 + 393 + 241) (9,498-9,954, 95% C.I.). As the fraction hereof found by a stringent fracture definition using a combination of both ICD-10 and NOMESCO coding is 9,092/9.726 = 93.5% (93.0-94.0% - 95% C.I.), the underestimation by this method is 6.5% (6.0-7.0%).

## Discussion

Our study showed a high accuracy of hip fractures registered in NPR when defined by a combination of ICD-10 and NOMESCO codes, excluding no more than 6.5% (6.0-7.0%) of all hip fractures defined by wider fracture-definitions.

A feasible method to monitor time trends in hip fracture incidence is warranted due to the high morbidity and mortality in this patient-group. The validity of data in NPR is also of value for the correct economic monetary transactions from national health budgets to hospital budgets, benchmarking between hospitals regarding effectiveness and adjustments of reimbursements based on Diagnosis related Group-systems. Furthermore, a WHO initiated initiative recently led to the development of the Fracture Risk Assessment Tool (FRAX®) allowing prediction of the 10-year probability of hip- and major osteoporotic fractures [18]. The tool is country-specific as fracture-rates in a global perspective are known to vary more than a 10-fold between different countries. A country-specific tool is now also available for Norway but has not been independently validated in Norway, as nationwide validated hip fracture incidence rates until now have been lacking. Therefore, an easily accessible hip fracture definition is important for monitoring of incidence- and prevalence rates and their time-dependent changes and thus monitoring public health. Our study showed a high accuracy of hip fractures registered in NPR when defined by a combination of ICD-10 and NOMESCO codes, excluding no more than 6.5% (6.0-7.0%) of all hip fractures defined by wider fracture-definitions.

### Frequency of coding errors in relation to definitions

In theory, definition of fractures by ICD alone could potentially be more correct, this would also include fractures missing NOMESCO coding, conservatively treated fractures as well as patients dying during their hospitalization for hip fracture (an estimated total of 321–491 fractures yearly). However, due to coding praxis in Norway, there is currently no way to isolate these conservatively treated fractures from irrelevant outpatient controls without examination of original health records. Even a restrictive definition of in-patients as admission for more than 24 hours would be inappropriate, as some conservatively treated fractures are quickly referred to rehabilitation centers, retirement homes or communal services. Using relevant NOMESCO coding as the sole discriminating value would also be inappropriate, as the overlap in coding between hip fractures and coxarthrosis-treated hip replacements is too high. Furthermore, the addition of a procedure code seems to validate ICD-codes as illustrated by the precision of fractures in the group defined by fracture- and procedure-code in combination – as well as the fact that only 12 further hip fractures (95% C.I. 1.9-6.5%) were found in the group defined by NOMESCO alone.

### Previous Norwegian and international hip fracture validation studies

Using our proposed stringent fracture definition, we found very good concordance between registered fractures in the NPR and true fractures with false positive rates of 0.6% and false negative rates of 6.5%, assuming close to complete coverage of NPR data as previously established in the NOREPOS study [19]. The NOREPOS study used an advanced algorithm in order to isolate incident hip fractures from NPR data combining ICD and NOMESCO in the time period 1999–2008 for all of Norway. Fractures were deemed not incident by procedure codes implying revision, secondary hospital stays or multiple records within a three week period and deemed possible by lacking procedure codes during the first hospital stay, rehabilitation as primary diagnosis code, performed hip arthroplasty or irrelevant procedure codes, respectively. A total of 27,274 out of 139,913 hospitalizations were classified as not incident, another 31,358 fractures were classified as possible whilst the rest were classified as incident fractures. The incident and possible fractures were compared to registered fractures in the Oslo Health Study from 2000–2001 as well as the Tromsø 4 study, including 295 and 732 fractures, respectively. For each of these studies, the NOREPOS algorithm showed false negative and false positive rates between 3 and 6.5%, equaling a combined Cohen’s kappa of 0.95.

Previously in Norway, local database validation studies have been performed by Grønskag *et al.*[20]*,* showing good validity of a combination of ICD and NOMESCO code. Lofthus *et al.*[11] and Emaus *et al.*[16] found overestimated fracture rates in NPR by ICD-10 code alone, with sizeable underestimation of incidence rates by a combination of ICD and NOMESCO due to missing procedure codes. In other countries with good locally or nationally defined background populations, Nymark *et al.*[7] found reduced sensitivity of a fracture definition by a combination of ICD and NOMESCO coding in Denmark. In Sweden, Finland and Canada, hip fracture incidence rates have been estimated both by ICD alone [21–25] or a combination of ICD and NOMESCO coding [26]. However, none of these studies validated their method of data extraction. In Canada, Lix *et al.*[27] compared fracture-incidence rates for Manitoba with fracture rates in the CaMOS study. Here, a combination of ICD and NOMESCO was most appropriate for men whilst the strict definition resulted in lower incidence rates for women. Using the Kaiser Permanente database, Huang *et al.*[28] found good concordance between ICD-9 coding for femoral neck and pertrochanteric fractures and radiology reports.

Thus, internationally there seems to be differences between reported findings, and one can speculate as to whether local coding practices are a driving factor for these findings. Ultimately, for Norway, our proposed method of monitoring fracture incidence rates seems to be no less accurate than other more time-demanding algorithms, and might also be workable in other countries.

### Other means of estimating hip fracture incidences in Scandinavia

Other databases or other approaches to monitor fractures incidences have been explored earlier, as described below.

Gjertsen *et al.*[9] used data from the Norwegian Hip Fracture Registry (NHFR), a non-compulsory database maintained by the Norwegian Orthopaedic Association, in which hip fractures defined by a combination of ICD-10 codes S72.0-2 and NOMESCO codes (NFBab/NFJab a,b = 0-9) were compared to similar data from NPR. On the one hand, the NHFR specifies implant failure as a separate entity, increasing the specificity of the registered data, whilst on the other hand it excludes non-surgically treated fractures, thus decreasing sensitivity. Gjertsen *et al.*[9] identified only 79% of fractures registered in NPR in the NHFR. In comparison, a similar database-comparison in Denmark by Nymark *et al.*[7] equaled an inter-database fracture consistency of 89.8%.

Finally, Joakimsen *et al.*[10] validated a non-database approach through questionnaire-based self-reporting of fractures as well as a computerized radiographic register for identifying incident fractures. Using manual inspection of case files, they found a 3–21% over-reporting by ICD-9 coding alone (820.0-9), with equal or worse results by the use of self-reporting or radiographic register.

The results of these methods seem inferior to our method of hip fracture incidence-estimation.

### A workable definition of hip fractures from databases

It is evident that regional differences in coding praxis exist even in recent materials. Thus, our own data found a low validity of ICD-coding alone for defining new incident fractures, as postoperative out-patient follow-up visits were also coded with the same primary ICD-code as fracture cases. Data from Grønskag *et al.*[20] from Norway and from Canada by Lix *et al.*[27] seem to support the notion of a strict combination of ICD and NOMESCO coding for fracture definition, while Nymark *et al.*[7] found suboptimal sensitivity for the identification of fractures by this definition, and other datasets, not directly validated, suggest the use of ICD alone as best measure. The present study shows that the underestimation of fracture incidence by strict combination of diagnostic and procedural codes is rather small. More elaborate algorithms used to estimate fracture incidences as in the NOREPOS study [19] does not seem to perform significantly better.

### Estimation of true fracture incidence

Using this definition, hip fracture incidence-rates are likely to deviate no more than 6.5% (6.0-7.0%) from true incidence rates. The study confirms that the true incidence rate of hip fractures of 189.4:100.000 person years in amongst the highest reported from any country.

### Strengths

Our study has several strengths. First, the nationwide approach levels out the effect of regional differences in coding practice or incidence rates. Second, the Norwegian health care system is public and tax-funded. Due to the pain and loss of function of hip fracture and tax- funding of the health care system, virtually all hip fracture cases are admitted to hospitals feeding data to the national registries. In Norway, diagnostic coding by the ICD system and concurrent procedure-codes classified by NOMESCO have been standardized since 1999 [6]. Generally, epidemiologic data from the Scandinavian countries are regarded to be very reliable [29]. Indeed, almost all (96.9%) of all treatments registered with ICD-code could be assigned to a civil personal registration number [30], so completeness of records can be assumed to be close to perfect.

### Limitations

Our study has a few weaknesses. First, health records were evaluated without review of X-ray images and relied on written reports. These were, however, usually performed by experienced radiologists and orthopedic surgeons, minimizing risk of bias. Fractures coded in NPR relies on coding by clinical departments rather than radiological findings, so discrepant records with radiologically described fractures without clinically defined fractures were not included. The agreement on concordance between X-ray reports and discharge summaries was considered straight forward and therefore done only by MH without analysis of interrater-reliability.

Secondly, only 80% of requested health records were available for examination, but analysis for non-response bias using available parameters from NPR (age of patient, gender and subsample-group of records) was performed without indication of underlying bias.

Thirdly, independent established coverage of NPR data was not performed. Estimation of true hip fracture incidence is based on both accuracy of data registered in NPR as well as coverage or completeness of event registration in NPR. In this study, no solitary assessment of coverage of NPR was performed, as this recently has been performed on a large scale by Omsland et al., using prospective data from the Oslo Health Studies and the Tromsø study, showing a combined Cohen’s kappa of 0.95 [19]. Furthermore, prospective identification of fractures over a two year period for the hospital of Southern Norway found no missing fractures in the ICD-10 coded register for the hospital, a register which amongst others forms the base of registrations in NPR [13]. Thus, the validity of the data in NPR is based upon our own accuracy studies and three separate regional examinations of coverage of NPR that show good coverage.

## Conclusion

This nation-wide, population based historical cohort of hip fractures drawn from the NPR showed a high accuracy of hip fracture identification when defined by an easily accessible combination of ICD-10 and NOMESCO codes, excluding no more than 6.5% of all hip fractures defined by wider fracture-definitions. Assuming close to perfect coverage of NPR data as previously established by the NOREPOS study [19], we propose the rather accurate and easily accessible combination of ICD and NOMESCO coding as a workable definition of hip fracture. Due to its ease, this method may be used for surveillance of hip fracture incidence evolution in the future.

## Electronic supplementary material

Additional file 1:ICD-10 DRG codes and NOMESCO procedure codes used in this study.(DOCX 11 KB)

Below are the links to the authors’ original submitted files for images.

Authors’ original file for figure 1

Authors’ original file for figure 2
