# Supplementary material for: The incidence of hip fractures in Norway –accuracy of the national Norwegian patient registry
Source: BMC Musculoskelet Disord. 2014 Nov 13;15:372. doi: 10.1186/1471-2474-15-372 (PMC4247646; doi:10.1186/1471-2474-15-372)
Supplement: Supplementary file 1 — Additional file 1:ICD-10 DRG codes and NOMESCO procedure codes used in this study.(DOCX 11 KB) [file 12891_2014_2321_MOESM1_ESM.docx]

Additional file: ICD-10 DRG codes and NOMESCO procedure codes used in this study

ICD-10 Codes used:

S72.0 Fracture of neck of femur

S 72.1 Pertrochanteric fracture

S 72.2 Subtrochanteric fracture

NOMESCO procedure version 1.14 codes used

NFB Primary prosthetic replacement of hip joint

NFB 0y primary partial prosthetic replacement of hip joint not using cement

NFB 1y primary partial prosthetic replacement of hip joint using cement

NFB 20 primary total prosthetic replacement of hip joint not using cement

NFB 30 primary total prosthetic replacement of hip joint using hybrid technique

NFB40 primary total prosthetic replacement of hip joint using cement

NFB 62 Primary prosthetic replacement of joint surface of femoral head

y=0: all parts, y=1: single part – distal, y=2: single part – proximal

NFJ Fracture surgery of femur

NFJ 0y Closed reduction of fracture of femur

NFJ 1y Open reduction of fracture of femur

NFJ 2y External fixation of fracture of femur

NJF 3y Internal fixation of fracture of femur using bioimplant

NFJ 4y Internal fixation of fracture of femur using wire, rod, cerclage or pin

NFJ 5y Internal fixation of fracture of femur using intramedullary nail

NFJ 6y Internal fixation of fracture of femur using plate and screws

NFJ 7y Internal fixation of fracture of femur using screws alone

NFJ 8y Internal fixation of fracture of femur using other or combined methods

NFJ 9y Other fracture surgery of femur

y=0: neck of femur, y=1: pertrochanteric, y=2: subtrochanteric
