# Supplementary material for: The incidence of hip fractures in Norway –accuracy of the national Norwegian patient registry
Source: BMC Musculoskelet Disord. 2014 Nov 13;15:372. doi: 10.1186/1471-2474-15-372 (PMC4247646; doi:10.1186/1471-2474-15-372)
Supplement: Supplementary file 2 — Authors’ original file for figure 1 [file 12891_2014_2321_MOESM2_ESM.pdf]

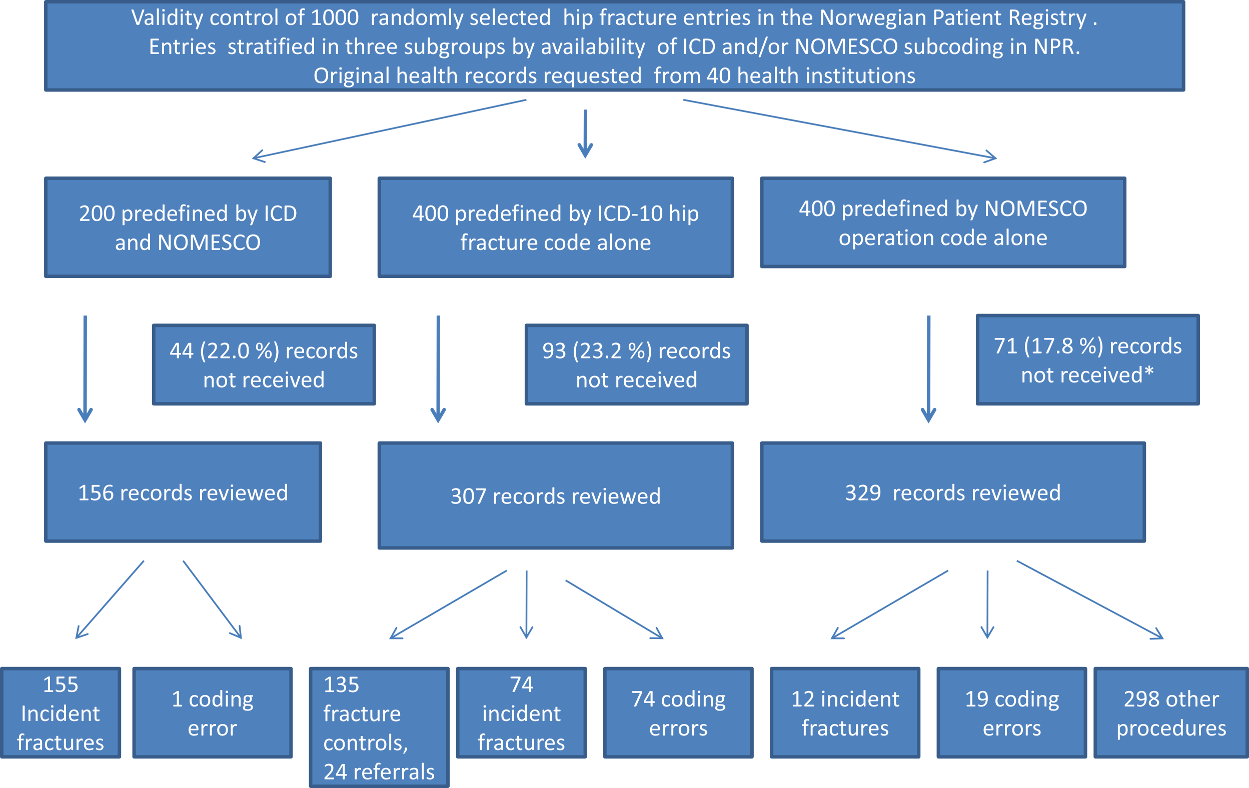
Validity control of 1000 randomly selected hip fracture entries in the Norwegian Patient Registry . Entries stratified in three subgroups by availability of ICD and/or NOMESCO subcoding in NPR. Original health records requested from 40 health institutions
200 predefined by ICD and NOMESCO
400 predefined by ICD-10 hip fracture code alone
400 predefined by NOMESCO operation code alone
44 (22.0 %) records not received
93 (23.2 %) records not received
71 (17.8 %) records not received*
156 records reviewed
307 records reviewed
329 records reviewed
155 Incident fractures
1 coding error
135 fracture controls, 24 referrals
74 incident fractures
74 coding errors
12 incident fractures
19 coding errors
298 other procedures
