# Supplementary figures and images for: The incidence of hip fractures in Norway –accuracy of the national Norwegian patient registry
Source: BMC Musculoskelet Disord. 2014 Nov 13;15:372. doi: 10.1186/1471-2474-15-372 (PMC4247646; doi:10.1186/1471-2474-15-372)

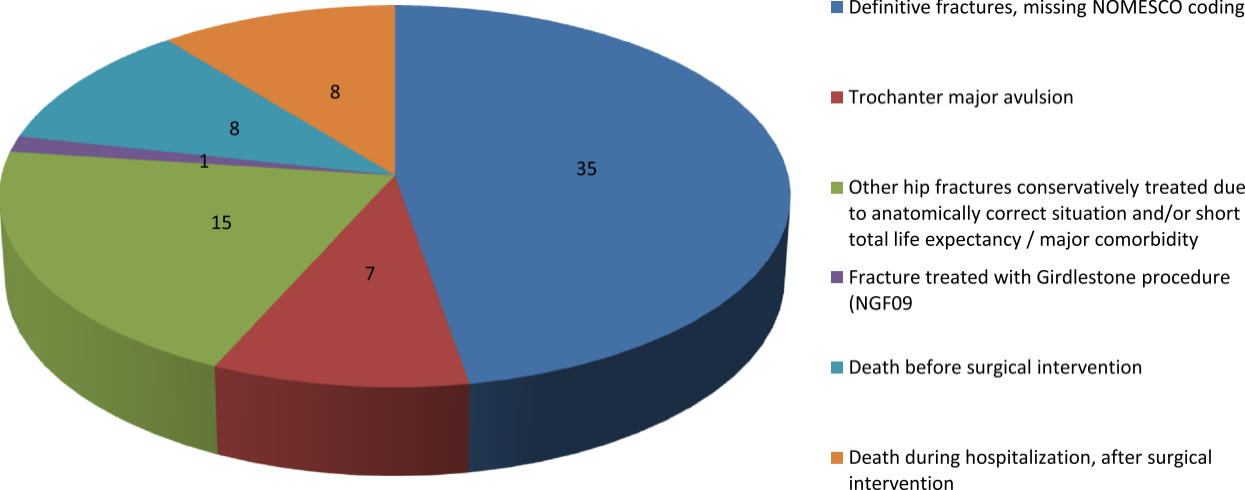

Supplement: Supplementary file 3 — Authors’ original file for figure 2 [file 12891_2014_2321_MOESM3_ESM.pdf]
